# Focusing on the premature death of redeployed miners in China: an analysis of cause-of-death information from non-communicable diseases

**DOI:** 10.1186/s12992-019-0450-5

**Published:** 2019-01-22

**Authors:** Wei Xian, Bing Han, Leizhen Xia, Yining Ma, Haodi Xu, Lu Zhang, Li Li, Hongbo Liu

**Affiliations:** 1 School of Public Health, China Medical University, Shenyang, People’s Republic of China; 2 Division of Environmental and Occupational Health Sciences, National Jewish Health, Denver, CO USA

**Keywords:** Redeployed miners, Non-communicable diseases, Premature death, Year of life lost

## Abstract

**Background:**

Reducing premature deaths is an important step towards achieving the World Health Organization’s sustainable development goal. Redeployed miners are more prone to disease or premature death due to the special occupational characteristics. Our aims were to describe the deaths of redeployed miners, assess the losses due to premature death and identify their main health problems. All the records of individuals were obtained from Fuxin Mining Area Social Security Administration Center. Year of life lost (YLL) and average year of life lost were used to assess the loss due to premature death. YLL rates per 1000 individuals were considered to compare deaths from different populations.

**Results:**

Circulatory system diseases contributed the most years of life lost in the causes of death, followed by neoplasms. But average year of life lost in neoplasms was 6.85, higher than circulatory system diseases, 5.63. Cerebrovascular disease and ischemic heart disease were the main causes of death in circulatory system diseases. And average years of life lost in cerebrovascular disease and ischemic heart disease were 5.85 and 5.62, higher than those in other circulatory system diseases. Lung cancer was the principal cause of death in neoplasms. Average year of life lost in liver cancer was 7.92, the highest in neoplasms.

**Conclusions:**

For redeployed miners, YLL rates per 1000 individuals in cerebrovascular disease, ischemic heart disease and lung cancer were higher than those in other populations, especially in men. It is important to attach importance to the health of redeployed miners, take appropriate measures to reduce premature death and achieve the sustainable development goal. Our findings also contribute to a certain theoretical reference for other countries that face or will face the same problem.

**Electronic supplementary material:**

The online version of this article (10.1186/s12992-019-0450-5) contains supplementary material, which is available to authorized users.

## Background

Although World Health Organization (WHO) has reported an upward trend in average life expectancy at birth, some populations still have premature deaths and premature mortality does not significantly decline. So it is difficult to achieve the WHO’s sustainable development goal of reducing by one-third premature mortality from non-communicable diseases (NCDs) through prevention and treatment by 2030. Therefore, an effective strategy to reduce premature death is to pay more attention to improve the health of populations who die prematurely.

Nowadays, a new crowd of people has emerged — redeployed miners, referring to miners who stopped working in the mining industry due to resource exhaustion [1] and no longer returned to mines again after being released. Unfortunately, the number of the redeployed miners will continue to increase because two-thirds of the resource-based mines and enterprises are faced with exhausted resource according to the report from the ministry of land and resources of China [2]. Due to the non-renewable characteristics of mineral resources, not only is China facing the problem of increasing redeployed miners, but also more and more countries around the world are facing or will face the same problems as China.

The redeployed miners had been exposed to harmful substances before being released from mines. Poor working conditions, including dust, humidity, vibration and noise, make it easy for miners to have non-communicable diseases, such as respiratory diseases, hypertension, stomach disorders, and so on [3–5]. Miners, especially underground miners, were also vulnerable to pneumoconiosis [6]. Therefore, their overall health is generally poor [7–9], making them more prone to disease or premature death. They may become populations who have low health level or premature deaths in the future, which directly affect overall health status in many countries around the world. It is crucial and imperative for redeployed miners to get more public attention.

Year of life lost (YLL) is used to measure losses of life due to premature death [10]. It differs from describing the number and proportion of deaths in traditional epidemiologic measures. YLL needs detailed death information to provide the data for calculation. So it is more fully reflecting the deaths occurring at younger ages in some cases [11] and the enormous economic loss due to premature death. It can be used to compare different causes of death and identify the main diseases that we need to prevent and control [12]. From a public health perspective, the study of YLL in special groups is fundamental and necessary to reduce the impact of premature death through the implementation of related public health policies.

The Fuxin Mining Industry Group has been a state-owned enterprise for over 100 years [13]. From 2003 to 2014, several mines of the Fuxin Mining Industry Group had been exhausted, and about thirty thousand redeployed miners were displaced from the mining industry. But what are the underlying causes of death in these miners? Are the effects of diseases different from those of the general population? Are miners the population who will die prematurely in the future? Therefore, describing the deaths of redeployed miners, assessing the losses due to premature death and identifying their main health problems are very important facts to explore. This helps in taking measures to reduce premature death and achieve the sustainable development goal.

## Methods

### Study populations

The participants in our study were from the Fuxin Mining Industry Group in a total of twenty mines, which have been gradually resource-exhausted since 2003. Presently, all the mines have stopped working. All enrolled individuals were redeployed miners from the Fuxin Mining Industry Group after 2003.

### Data source

All individuals were investigated at the Fuxin Mining Area Social Security Administration Center, including gender, date of birth, workplaces, type of work and current status (alive or deceased). By the end of 2014, we collected deceased redeployed miners’ death data based on the death certificates, mainly including gender, date of birth, date of death, workplaces, major occupation and type of work, place of death, cause of death, etc.

### Methodology for coding the causes of death

We coded the causes of death by International Classification of Diseases-10th revision (ICD-10). According to ICD-10, the underlying causes of death were divided into 15 categories, including: I. Certain infectious and parasitic diseases (A00-B99); II. Neoplasms (C00-D48); III. Diseases of the blood and blood-forming organs, and certain disorders involving the immune mechanisms (D50-D89); IV. Endocrine, nutritional and metabolic diseases (E00-E90); V. Mental and behavioral disorders (F00-F99); VI. Nervous system diseases (G00-G99); VII. Circulatory system diseases (I00-I99); VIII. Respiratory diseases (J00-J99); IX. Digestive system diseases (K00-K93); X. Diseases of the skin and subcutaneous tissue (L00-L99); XI. Musculoskeletal system and connective tissue diseases (M00-M99); XII. Genitourinary system diseases (N00-N99); XIII. Deformity/distortion and chromosome abnormality (Q00-Q99); XIV. External causes of diseases and deaths (V01-Y98); XV. Symptoms, signs and abnormal clinical and laboratory findings, not elsewhere classified (R00-R99).

If only one cause of death was recorded on death certificate, we chose it as the underlying cause. If multiple causes of death were recorded, we needed to determine which one is the underlying cause. In the coding process, we searched the literature to determine the cause of death without exact ICD code [14]. When it was not found in the literature, we resorted to use medical knowledge to determine whether the discrepancies were due to translation or a disease with different names. Some causes of death were eventually coded by considering the progression of their diseases.

### Statistical analysis

The statistical analyses were conducted in SAS software, version 9.4. We calculated the premature mortality of the redeployed miners, described their deaths and the distribution of the main causes of death. Age of death was the difference between the date of birth and death. Premature mortality was the probability of dying between the ages of 30 and 70 years. Year of life lost (YLL) and average year of life lost (AYLL) were used to assess the loss due to premature death. AYLL was simply an average derived by dividing the total YLL by the number of disease deaths. We additionally considered YLL rates per 1000 individuals to compare deaths from different populations. YLL rates per 1000 individuals were calculated by (YLL/n) × 1000, where n is the number of all miners.

The initial formula calculated YLL without discounting or age weights:

1 $$ \mathrm{YLL}=\mathrm{N}\ast \mathrm{L}. $$

The formula calculated YLL with 3% discounting and uniform age weights:

2 $$ \mathrm{YLL}=\frac{\mathrm{N}}{0.03}\left(1-{e}^{-0.03L}\right). $$

We considered both non-zero discounting and age weighting to calculate YLL. So the full formula is as follows [15]:

3 $$ \mathrm{YLL}=\mathrm{N}\frac{Ce^{(ra)}}{{\left(\beta +r\right)}^2}\left[{e}^{-\left(\beta +r\right)\left(L+a\right)}\left[-\left(\upbeta +\mathrm{r}\right)\left(\mathrm{L}+\mathrm{a}\right)-1\right]-{e}^{-\left(\beta +r\right)a}\ \left[-\left(\upbeta +\mathrm{r}\right)\mathrm{a}-1\right]\right]. $$

Where:

- N: Number of the deaths at a certain age and gender.
- L: The loss of standard life expectancy at that age and gender.
- a: Age at death.
- r: Discount rate.
- β: A parameter of the age-weighting function.
- C: Constant number.

In accordance with the standard approach of Global Burden of Disease (GBD), C is the age-weighting correction constant recommended by the World Bank, equal to a value of 0.1658. β is a parameter of the age-weighting function, equal to 0.04, which controls the shape of function [16]. The discount rate is 3%, which is the GBD standard value. To compare with other studies, the calculation of YLLs used the life tables provided in the GBD Study, the standard model life table, West Level 26 [17]. In this table, life expectancy at birth for women and men are 82.5 and 80, respectively.

In the GBD study, an age-weighting factor in YLL reflects the fact that in most societies, young people are usually endowed with higher social values. In addition, most people tend to favor current benefits rather than future ones, so the discount rate (social time preference) is included in YLL. YLL is more reasonable given the relative importance of age (age weight) and time (discount rate).

## Results

### The basic situation of death

Seven thousand three hundred twelve of the redeployed miners from Fuxin city had died between 2003 and 2014. The average age of death was 73.08 (95% CI 72.88 to 73.29). For redeployed miners, premature mortality from NCDs was 29.58% and premature mortality from the four main NCDs (cardiovascular diseases, cancer, diabetes and chronic respiratory diseases) was 30.30% in 2003–2014. Furthermore, premature mortality from the four main NCDs was 24.85% in 2012, which was higher than that of China reported by the WHO in the same year, 19.4% [18]. Death from different age groups and genders was shown in Table 1.

**Table 1:** Distribution of death population by different age groups and genders, 2003–2014

Age group	Man		Woman		Total	
	Number of deaths	Proportion (%)	Number of deaths	Proportion (%)	Number of deaths	Proportion (%)
< 45	20	0.27	1	0.01	21	0.28
45~49	30	0.41	11	0.15	41	0.56
50~54	214	2.93	21	0.29	235	3.22
55~59	367	5.02	24	0.33	391	5.35
60~64	454	6.21	14	0.19	468	6.40
65~69	964	13.18	35	0.48	999	13.66
70~74	1644	22.48	41	0.56	1685	23.04
75~79	1721	23.54	23	0.31	1744	23.85
80~84	1140	15.59	14	0.19	1154	15.78
85~89	438	5.99	5	0.07	443	6.06
≥90	130	1.78	1	0.01	131	1.79
Total	7122	97.40	190	2.60	7312	100

### YLLs and economic losses due to premature death

Among the 7312 death population, 165 miners had uncertain causes of death. There were 20 individuals who only had symptoms, signs, clinical and laboratory’s abnormalities. Their causes of death were marked as R00-R99. Forty-one miners had certain infectious and parasitic diseases. Different causes of death from NCDs in the rest of 7086 people are shown in Table 2. Circulatory system diseases contributed the most YLLs, followed by neoplasms and respiratory diseases. However, AYLL in neoplasms was 6.85, higher than circulatory system diseases, 5.63 and respiratory diseases, 4.66.

**Table 2:** Years of life lost (YLLs) by various causes of death from NCDs, 2003–2014

Causes of death	Man		Woman		Total		Rank
	N	YLLs	N	YLLs	N	YLLs	
Circulatory system diseases	4166	23,471.78	97	822.11	4263	24,293.89	1
Neoplasms	1678	11,489.23	56	594.06	1734	12,083.29	2
Respiratory diseases	423	1970.95	7	57.91	430	2028.86	3
External causes of diseases and deaths	126	1203.21	4	54.62	130	1257.83	4
Digestive system diseases	159	1136.69	6	68.87	165	1205.56	5
Endocrine, nutritional and metabolic diseases	166	1011.52	6	61.56	172	1073.08	6
Nervous system diseases	94	462.70	4	26.63	98	489.33	7
Genitourinary system diseases	63	379.62	4	49.93	67	429.55	8
Other diseases^a^	26	162.92	1	7.48	27	170.4	–
Total	6901	41,288.62	185	1743.17	7086	43,031.79	–

^a^Other diseases include musculoskeletal system and connective tissue diseases, diseases of Blood, blood-forming organs and immune mechanisms, mental and behavior disorders, diseases of the skin and subcutaneous tissue and deformity/distortion and chromosome abnormality

We are more concerned with diseases that contribute more YLLs and more deaths—circulatory system diseases and neoplasms (Table 3). Cerebrovascular disease and ischemic heart disease were the main causes of death in circulatory system diseases. AYLLs in cerebrovascular disease and ischemic heart disease were 5.85 and 5.62, higher than those in other circulatory system diseases. YLLs were the highest in lung cancer, followed by esophagus cancer, liver cancer and stomach cancer, accounting for over 69% of the total YLLs in neoplasms. AYLL in liver cancer was 7.92, the highest in neoplasms. We chose published articles [19–21], which calculated YLLs in different populations and used the same statistical methods as ours. For redeployed miners, YLL rates per 1000 individuals in circulatory system diseases and neoplasms were higher than those in other populations.

**Table 3:** YLLs and economic losses of circulatory system diseases and neoplasms, 2003–2014

Causes of death		N	YLLs	Economic losses (USD)	YLL rates per 1000 individuals		
					This study	Reference 1^a^	Reference 2
Circulatory system diseases	Cerebrovascular disease	2476	14,495.10	7,162,317.49	35.20	–	7.36^c^
	Ischemic heart disease	1164	6544.24	3,412,189.33	15.89	6.40	3.00^b^
	Other types of heart disease	398	2105.21	1,319,862.60	5.11	–	–
	Pulmonary heart disease and pulmonary circulation disease	159	727.07	288,102.37	1.77	–	–
	Hypertension	48	300.38	155,289.07	0.73	–	–
Neoplasms	Lung cancer	530	3569.89	2,143,618.63	8.67	3.60	5.60^b^
	Esophagus cancer	273	2008.82	1,503,522.32	4.88	–	0.58^c^
	Liver cancer	183	1450.19	1,133,565.54	3.52	2.60	3.30^b^
	Stomach cancer	212	1347.63	709,782.18	3.27	1.80	1.67^c^
	Intestinal cancer	122	807.78	534,412.16	1.96	–	–
	Pancreatic (head) cancer	70	478.58	265,982.96	1.16	–	–
	Urinary bladder cancer	45	215.96	65,470.99	0.52	–	–
	Laryngeal cancer	23	159.34	163,874.08	0.39	–	–

^a^Reference 1 was from the published article [19]

^b^The data was from the published article [20]

^c^The data was calculated based on the numerical results provided in the published article [21]

Table 4 lists the results of YLL rates in our study and Hubei Province, which is dominated by heavy industry. YLL rates in the urban areas of the Hubei province were further processed according to the data provided in the article [21]. The results showed that YLL rates among men were prominently higher in our study. YLL rates in cerebrovascular disease, ischemic heart disease and lung cancer among women were higher. YLL rates in esophageal and gastric cancer were lower, but the difference was not significant.

**Table 4:** YLL rates per 1000 individuals in our study and Hubei Province

Causes of death	Man		Woman	
	This study	Reference^a^	This study	Reference^a^
Cerebrovascular disease	42.88	10.37	5.34	4.28
Ischemic heart disease	19.13	7.04	3.32	1.46
Lung cancer	10.22	6.31	2.65	1.86
Esophagus cancer	6.11	1.00	0.10	0.15
Stomach cancer	3.98	2.43	0.51	0.90

^a^The data was calculated based on the numerical results provided in the published article [21]

## Discussion

With the development of economy and society and the continuous improvement of medical conditions, people’s living standards and quality have been continuously improved. In recent years, life expectancy has been increasing. Reducing and preventing the premature death of redeployed miners due to occupational hazards is becoming more and more important. In our study, premature mortality from the main NCDs was higher among redeployed miners than the overall Chinese population [18], indicating that redeployed miners were high-risk populations who died prematurely. In order to better achieve the sustainable development goal, we should prevent the diseases having higher YLLs and reduce the losses of these diseases due to premature death.

YLLs in circulatory system diseases and neoplasms in our study were higher than other causes of death in any year (see Additional file 1). As expected, circulatory system diseases, neoplasms and respiratory diseases were the main causes of death, regardless of taking into account rates of death or YLLs. Causes of death are inseparable from the bad working conditions of the mines. Because the mining job is a special profession, fewer women were working in the mines, especially underground work. The severity and impact of occupational hazards is different between men and women. Our results also showed that YLLs in men were higher than in women and YLLs in some diseases among women were 0. Men were at higher occupational risk than women. Cerebrovascular disease and ischemic heart disease were extremely major causes of death in circulatory system diseases. Working in wet and noisy environments for a long time is more likely to lead to a miner’s hypertension [22]. And hypertension was the main cause of cerebrovascular disease [23]. For redeployed miners, YLL rates per 1000 individuals in cerebrovascular disease and ischemic heart disease were higher than those in other populations. The reasons may be that the increased risk of death from ischemic heart disease with continuous exposure to dust [24]. The difference had existed in both men and women.

The basic population of Fuxin City is similar to urban areas of Hubei Province. The time was also included in the time frame of our study. Also, the same statistical method was used in both studies, so we chose urban areas of Hubei Province as a reference. YLL rates per 1000 individuals in lung cancer were higher among both men and women than in the urban areas of the Hubei province. This suggested that lung cancer is the leading cause of death in neoplasms, whether in man or woman. Although miners have left the dusty working environment after their redeployment, the residual dust in the lungs remains difficult to remove. The residual dust in the lungs and the history of dust exposure affect the health of the miners, making them more prone to pneumoconiosis and a higher risk of cancer [25, 26]. Many hazardous substances in the environment can make miners more susceptible to lung diseases than the general population [27]. YLLs in esophagus cancer, liver cancer and stomach cancer were also higher. It is possible that the pressure and irregular eating diet among miners lead to the relatively high incidences of digestive system cancer. YLL rates among women were not higher than that in the urban areas of the Hubei province. It may again be attributed to dominantly more male miners working in underground mining.

As global coal resources continue to decrease, more and more coal mines will be shut down because of exhausted resource in the future, which will result in a large number of redeployed miners. If the death of the general population is used as a reference, it does not reflect the increase in premature deaths and mortality due to occupational diseases [28, 29]. Therefore, our findings can contribute to a certain theoretical basis and reference for other countries in the world that face or will face the same problem. At present, the public are paying more and more attention to the health and work protection of actively employed miners. For redeployed miners, they are often neglected and have no relevant policies or measures to better protect their health. Therefore, more attentions and actions are needed now. We hold that the allocation of health resources should be tilted in a certain extent toward redeployed miners, paying more attention to lung cancer, cerebrovascular disease, and ischemic heart disease. One suggestion is to set up a free medical examination center for them. The check should focus more on pulmonary function and cardiovascular or cerebrovascular functions. Another suggestion is to provide them with medical assistance to reduce premature deaths and achieve the sustainable development goal.

There are some limitations in our study. Fuxin City is the first resource-exhausted city in China. There are a large number of redeployed miners there. Therefore, we used the data of redeployed miners in Fuxin City as a representative of this population. Our study focuses mainly on calculating the loss due to premature death and on the difference in YLLs between redeployed miners and other populations. Death-related variables (smoking history, disease histories, etc.) were not analyzed. The impact of death-related variables may be studied in future studies.

## Conclusions

Circulatory system diseases and neoplasms were the main causes of premature death among redeployed miners. Cerebrovascular disease and lung cancer were the dominant causes of death in circulatory system diseases and neoplasms, respectively. For redeployed miners, YLL rates per 1000 individuals in cerebrovascular disease, ischemic heart disease and lung cancer were higher than those in other populations, especially in men. It indicated that the special working environment and occupational characteristics have potentially adverse effects on the health of redeployed miners. It is very significant for achieving the sustainable development goal to attach importance to the health of redeployed miners and take appropriate measures to reduce premature death. Our findings also contribute to a certain theoretical basis and reference for other countries in the world that face or will face the same problem.

## Additional file

Additional file 1: YLLs by various causes of death from NCDs, 2003–2014. It is an additional table that supports the discussion section. (DOCX 21 kb)

## Abbreviations

AYLL: Average year of life lost; GBD: Global Burden of Disease
ICD-10: International Classification of Diseases-10th revision
NCDs: Non-communicable diseases
WHO: World Health Organization
YLL: Year of life lost
