# Supplementary material for: Focusing on the premature death of redeployed miners in China: an analysis of cause-of-death information from non-communicable diseases
Source: Global Health. 2019 Jan 22;15:7. doi: 10.1186/s12992-019-0450-5 (PMC6341550; doi:10.1186/s12992-019-0450-5)
Supplement: Supplementary file 1 — YLLs by various causes of death from NCDs, 2003–2014. It is an additional table that supports the discussion section. (DOCX 21 kb) [file 12992_2019_450_MOESM1_ESM.docx]

Additional file 1: **Table S1.** YLLs by various causes of death from NCDs, 2003-2014

| Death causes | 2003 | 2004 | 2005 | 2006 | 2007 | 2008 | 2009 | 2010 | 2011 | 2012 | 2013 | 2014 |
| --- | --- | --- | --- | --- | --- | --- | --- | --- | --- | --- | --- | --- |
| Cancer(C00-D48) | 7.57 | 403.17 | 575.88 | 682.75 | 954.28 | 1559.96 | 1490.04 | 1666.93 | 1431.18 | 1198.01 | 1306.49 | 807.00 |
| Diseases of Blood,  hematopoietic organs and immune mechanisms(D50-D89) | - | 7.13 | 9.60 | - | - | 6.69 | - | 17.94 | 4.51 | - | 2.56 | - |
| Endocrine, nutritional and metabolic diseases(E00-E90) | - | 12.64 | - | 73.86 | 65.81 | 94.46 | 133.65 | 138.81 | 111.30 | 156.75 | 192.79 | 93.02 |
| Mental and behavior  barrier(F00- F99) | - | - | - | 8.63 | 11.94 | - | 12.63 | 8.30 | - | - | - | - |
| Nervous system diseases(G00-G99) | - | 4.77 | 48.16 | 43.87 | 50.76 | 50.06 | 47.51 | 77.52 | 57.64 | 39.20 | 27.38 | 42.46 |
| Circulation system diseases(I00-I99) | 35.42 | 864.82 | 1481.24 | 1349.82 | 2576.57 | 2865.44 | 2755.42 | 2616.71 | 2918.06 | 2734.56 | 2618.00 | 1477.83 |
| Respiratory diseases(J00-J99) | 3.85 | 77.96 | 90.61 | 147.90 | 199.43 | 242.82 | 183.77 | 246.08 | 234.29 | 199.26 | 247.67 | 155.21 |
| Digestive system diseases(K00-K93) | - | 22.85 | 27.44 | 58.21 | 119.31 | 109.11 | 86.11 | 195.15 | 213.23 | 134.63 | 174.93 | 64.58 |
| Diseases of skin and subcutaneous tissue(L00-L99) | - | - | - | - | - | 12.55 | - | - | - | - | - | - |
| Musculoskeletal system and connective tissue diseases(M00- M99) | - | - | 4.15 | - | - | 37.29 | - | 8.48 | - | 7.34 | - | 5.14 |
| Genitourinary system diseases(N00-N99) | 4.77 | 17.69 | 16.81 | 19.26 | 73.99 | 60.85 | 86.08 | 36.98 | 24.77 | 48.66 | 18.10 | 21.60 |
| Deformity/distortion and chromosome abnormality  (Q00-Q99) | - | - | - | - | - | - | - | - | - | - | 5.52 | - |
| External factors of diseases and deaths(V01-Y98) | - | 56.62 | 133.26 | 154.46 | 35.68 | 163.79 | 155.23 | 171.85 | 136.06 | 134.43 | 83.73 | 32.73 |
| Total | 51.61 | 1467.65 | 2387.15 | 2538.76 | 4087.77 | 5203.02 | 4950.44 | 5184.75 | 5131.04 | 4652.84 | 4677.17 | 2699.57 |
